# The Nutritional Status and Eating Patterns of Female Secondary School Students in Kirkuk City, Iraq: A Cross-Sectional Study

**DOI:** 10.7759/cureus.66297

**Published:** 2024-08-06

**Authors:** Omed H Mehammed-Ameen, Marwah A Khalaf, Rabab H Hanoon

**Affiliations:** 1 Community Health Nursing, College of Nursing, University of Kirkuk, Kirkuk, IRQ; 2 Maternal and Child Health, College of Nursing, University of Kirkuk, Kirkuk, IRQ

**Keywords:** students, iraq, female, eating patterns, nutritional status

## Abstract

Background

Adolescents are increasingly facing nutrition-related issues such as overweight, obesity, and underweight, and eating patterns may significantly contribute to these issues. So, this study aims to assess the nutritional status and eating patterns of female secondary school students in Kirkuk City, Iraq.

Methods

A descriptive cross-sectional study design was applied, which took place between January 13 and May 30, 2024. The convenience non-probability sampling method was used to select 525 female secondary students in six female government secondary schools. Interview techniques and a structured questionnaire were used to collect data. IBM SPSS Statistics for Windows, Version 27.0 (Released 2020; IBM Corp., Armonk, New York, United States), was used for the data analysis.

Results

The mean age of the participants was 15.66 years old (SD 1.56). The finding revealed that 84 (16%) of secondary school female students were underweight, 100 (19%) of them were overweight, and 22 (4%) were obese. On the other hand, 423 (80.6%) of them reported eating snacks between meals, 497 (94%) reported eating fast food, and 378 (72%) reported skipping meals. The chi-squared test concludes that eating fast food and consuming energy drinks show significant associations with weight status, with a p-value less than 0.05.

Conclusion

A quarter of the female secondary school students were overweight or obese, and about one-sixth of them were underweight. Most of them either had snacks between meals, frequently ate fast food, or skipped meals. For these reasons, it is crucial to implement a school-based nutrition program in Iraqi schools.

## Introduction

Having a well-balanced and healthy diet is essential for maintaining resilience, promoting physical growth, enhancing cognitive development, and increasing productivity. Researchers recognize malnutrition as a significant issue that hinders the learning capacity of children and adolescents and results in lower achievement in school [1]. Globally, 10% of adolescents are overweight and 2-3% are obese. The prevalence of underweight among adolescent girls and males aged 15-19 years is 29% and 59%, respectively [2]. Currently, overweight/obesity ranks as the sixth most significant cause of death globally [3].

Adolescence is an important stage of growth and development as it marks the shift from childhood to adulthood, encompassing the age range of 10-19 years. During this critical phase, food patterns have a significant impact on long-term nutritional status and health [4,5]. The nutritional state during adolescence is a significant factor in the human lifecycle [6]. The worldwide increase in economic growth and urbanization has led to significant changes in the weight status of adolescents globally [7]. Developing countries have documented a decline in the prevalence of undernutrition. Conversely, there has been a growing trend of greater rates of overweight and obesity among teenagers in nations that are both developed and developing [8].

Following 2003, Iraq experienced progressive economic growth, leading to the adoption of a sedentary lifestyle and the eating habits of a diet that's high in fat and low in dietary fiber among people in general and especially teenagers, particularly in recent years [9]. On the other hand, skipping meals, especially breakfast, has become common among children and adolescents [10]. Certainly, the dietary habits of adolescents differ from those of children. The rising socioeconomic and academic requirements associated with this growth and developmental phase led to meal skipping, increased intake of processed and fast foods, and reduced consumption of vegetables and fruits [11]. Additionally, increased care given to body image and appearance by adolescent females negatively impacts their nutritional patterns. Dissatisfaction with body image, which refers to an adolescent female's feelings and reflections towards her body, has deteriorating nutritional consequences [12].

Adolescent surveys in Iraq reveal that the prevalence rates of overweight and obesity among adolescents aged 13-15 years in intermediate schools are 20.6% and 22.6%, respectively [13]. The availability of plentiful foods at affordable costs and alterations in societal perceptions of body image are contributing factors to the development of obesity [14]. Therefore, it is necessary to identify the factors that contribute to the abnormal nutrition status among adolescents. This involves investigating the consumption patterns of various types of food, including fast food (rich in fat and consumed quickly), beverages, coffee, high-sugar items, processed snacks, dining out, and the abandonment of traditional eating customs, such as eating alone. Investigating sedentary behaviors, such as prolonged sitting while interacting with media platforms like social media and video games, is also a crucial factor [15,16].

There is a prevalence of abnormal nutrition status and eating patterns among Iraqi adolescents, particularly female adolescents, and there are no specific studies on nutritional status, eating habits, or the association between socio-demographic characteristics and health and nutrition; hence, in order to fill this gap, this study was conducted. The aim of this cross-sectional study was to evaluate the nutritional status and eating patterns of female secondary students.

## Materials and methods

Design of the study

A quantitative descriptive cross-sectional study design was applied.

Setting and duration of the study

Six female government secondary schools from various districts in Kirkuk City, Iraq, were selected to conduct the study, which took place between January 13 and May 30, 2024.

Tool of the study

The researchers used an interview technique to collect data using a questionnaire that was developed after a thorough literature review. Firstly, a pilot study was conducted among 20 female secondary students to refine the instrument's contents, and a panel of four experts also revised it. Assessing the internal consistency of the instrument was conducted using Cronbach's alpha test, resulting in a total reliability of 0.81 [17]. The questionnaire consisted of three parts: Part 1, socio-demographic characteristics of the participants, which include age, height, weight, school grade, mother and father education and occupation, family size, and economic status; Part 2, a table of basic food group consumptions among female adolescents, including meat/alternatives, eggs, dairy, vegetables, fruits, breads/alternatives, rice, and other cereals; and Part 3, participants' eating habits and lifestyles encompassing aspects such as snacking, skipping meals, consuming fast food, consuming sweets, consuming hot and energy drinks, engaging in physical activity, mobile devices, and tablets, and the number of hours they sleep each day.

A retested scale was used to measure the participants' body weight to the nearest 0.5 kg. We achieved this by removing thick and heavy clothes. An accurate stadiometer was used to measure their height, and they stood upright without shoes, placed their heels together, ensured that their heels, buttocks, and back of head touched the upright rod, and recorded their height to the nearest 0.1 cm.

Body mass index (BMI) was determined by dividing the measured weight (in kilograms) by the square of the measured height (in square meters). BMI serves as an indicator of one's nutritional status. According to the classification by the World Health Organization (WHO), a BMI below 18.5 kg/m^2^ is considered underweight, between 18.5 and 24.9 kg/m^2^ is considered normal weight, from 25 to 29.9 kg/m^2^ is considered overweight, and 30.0 kg/m^2^ and beyond is considered obesity [18].

Sample and sampling

In six government secondary schools, we used convenience sampling methods (non-probability sampling) to select 525 female secondary students from grades 1 to 6 without affecting their class time. The minimum sample size calculation was done by the G*Power program to achieve adequate statistical power. We determined that a minimum sample size of 495 samples was required. This conclusion was derived based on a small effect size (0.2), a 95% confidence level, and a 5% margin of error [19].

Ethical consideration

The Research Ethics Committee at the College of Nursing, University of Kirkuk (No. 5), granted official approval on March 29, 2024. Other official approval was also obtained from the General Directorate of Kirkuk Education (No. 18290 on March 24, 2024). The managers and teachers of the participating school provided their consent and facilitated the data collection process. Teachers distributed written parental consent forms to students, requesting their consent for their child's involvement in the study.

Statistical analysis

We first entered the data directly from the pre-coded questionnaire into Excel spreadsheets. We conducted the statistical analysis using IBM SPSS Statistics for Windows, Version 27.0 (Released 2020; IBM Corp., Armonk, New York, United States). Descriptive statistics like frequency, percentage, mean, standard deviation, and cross-tabulation were used to present and summarize the data. The chi-squared test was conducted to identify associations between variables with a p-value less than 0.05. An ordinal logistic regression also was conducted to determine the effects of some variables on the participants' weight status with a 95% confidence level and p-value less than 0.05.

## Results

Two-thirds (66.1%) of the sample were between 15 and 17 years old, with an age mean score of 15.66 years. Students in the second and third grades constitute 42.9% of the sample. Regarding educational level, most mothers and fathers have diplomas and higher degrees (42.9% and 51.8%, respectively); 54.1% of mothers were housewives, and 63.8% of fathers were government employees; 69% of the sample's family size was between four and six members; 76.2 had a middle economic status; 61% had a normal weight; 16% were considered underweight; and 23% were overweight or obese (Table 1).

**Table 1:** Participants' socio-demographic characteristics (n=525) F: frequency; %: percentage

Items		F	%
Age group (in years)	12-14	144	27.4
	15-17	347	66.1
	≥18	34	6.5
Mean (15.66)/standard deviation (1.56)			
Grade	1-2	151	28.8
	3-4	225	42.9
	5-6	149	28.4
Mother's educational level	No formal education	60	11.4
	Primary	95	18.1
	Secondary	145	27.6
	Diploma and above	225	42.9
Father's educational level	No formal education	49	9.3
	Primary	53	10.1
	Secondary	151	28.8
	Diploma and above	272	51.8
Mother's occupation	Government employee	241	45.9
	Housewife	284	54.1
Father's occupation	Government employee	335	63.8
	Self-employee	190	36.2
Family size	<3	28	5.3
	4-6	362	69.0
	≥7	135	25.7
Economic status	High	107	20.4
	Middle	400	76.2
	Low	18	3.4
Weight status	Underweight	84	16
	Normal weight	319	61
	Overweight	100	19
	Obesity	22	4
Total		525	100

Table 2 reveals that 16% of secondary school female students were underweight, 19% were overweight, 4% were obese, and the remaining students were of normal weight (61%). The chi-squared statistical test, with a p-value less than 0.05, revealed a statistical association between nutrition status and age group, grade, and father's education level. On the other hand, the findings show no significance associated with mother's education level, mother's occupation, father's occupation, family size, and economic status. 

**Table 2:** Association between sample nutritional status and their socio-demographic variables F: frequency; %: percentage; *: statistically significant

Variable	Underweight		Normal		Overweight		Obese		Total		P-value
	F	%	F	%	F	%	F	%	F	%	
Age group (in years)											
12-14	39	46.4	82	25.7	22	22	1	4.5	144	27.4	<0.001^*^
15-17	44	52.4	213	66.8	69	69	21	95.5	347	66.1	
≥18	1	1.2	24	7.6	9	9	0	0	34	6.5	
School grade											
1-2	39	46.4	85	26.6	26	26	1	4.5	151	28.8	<0.001^*^
3-4	34	40.5	138	43.3	37	37	16	72.7	225	42.9	
5-6	11	13.1	96	30.1	37	37	5	22.7	149	28.4	
Mother's education											
No formal education	8	9.5	39	12.2	12	12	1	4.5	60	11.4	0.259
Primary	10	11.9	66	20.7	18	18	1	4.5	95	18.1	
Secondary	27	32.1	84	26.3	24	24	10	45.5	145	27.6	
Diploma and above	39	46.4	130	40.8	46	46	10	45.5	225	42.9	
Father's education											
No formal education	9	10.7	34	10.7	5	5	1	4.5	49	9.3	0.047^*^
Primary	4	4.8	35	11	12	12	2	9.1	53	10.1	
Secondary	22	26.2	87	27.3	29	29	13	59.1	151	28.8	
Diploma and above	49	58.3	163	51.1	54	54	6	27.3	272	51.8	
Mother's occupation											
Employee	44	52.4	133	41.7	51	51	13	59.1	241	45.9	0.099
Housewife	40	47.6	186	58.3	49	49	9	40.9	284	54.1	
Father's occupation											
Employee	54	64.3	207	64.9	60	60	14	63.6	335	63.8	0.850
Self-employee	30	35.7	112	35.1	40	40	8	36.4	190	36.2	
Family size number											
<3	6	7.1	12	3.8	6	6	4	18.2	28	5.3	0.081
4-6	61	72.6	218	68.3	69	69	14	63.6	362	69	
≥7	17	20.2	89	27.9	25	25	4	18.2	135	25.7	
Economic status											
Low	2	2.4	15	4.7	1	1	0	0	18	3.4	0.238
Middle	61	72.6	248	77.7	76	76	15	68.2	400	76.2	
High	21	25	56	17.6	23	23	7	31.8	107	20.4	
Total	84	16	319	61	100	19	22	4	525	100	

Table 3 shows the food types consumed by participants. The findings indicate that 25% of the participants never consume meat, 28.6% consume it rarely, 23.4% never eat eggs, and the consumption of bread, rice, and other cereals is high (76.6% and 73.1%, respectively). With a p-value less than 0.05, there is a significant statistical association between meat consumption, vegetable consumption, and weight status. On the other side, the table indicates that there is no significant association between weight status and other dietary consumption, including egg consumption, dairy consumption, fruit consumption, bread consumption, and rice and other cereal consumption.

**Table 3:** Association between participants' weight status and food type consumption F: frequency; %: percentage; *: statistically significant

Variable	Underweight		Normal		Overweight		Obese		Total		P-value
	F	%	F	%	F	%	F	%	F	%	
1. Meat											
Daily	13	15.5	17	5.3	6	6	0	0	36	6.9	0.002^*^
2-3 times/week	16	19	62	19.4	33	33	5	22.7	116	22.1	
Weekly	6	7.1	65	20.4	14	14	7	31.8	92	17.5	
Rarely	24	28.6	95	29.8	26	26	5	22.7	150	28.6	
Never	25	29.8	80	25.1	21	21	5	22.7	131	25	
2. Egg											
Daily	21	25	115	36.1	40	40	7	31.8	183	34.9	0.09
2-3 times/week	19	22.6	63	19.7	14	14	2	9.1	98	18.7	
Weekly	11	13.1	25	7.8	18	18	4	18.2	58	11	
Rarely	14	16.7	36	11.3	10	10	3	13.6	63	12	
Never	19	22.6	80	25.1	18	18	6	27.3	123	23.4	
3. Dairy											
Daily	42	50	162	50.8	55	55	14	63.6	273	52	0.922
2-3 times/week	18	21.4	70	21.9	19	19	4	18.2	111	21.1	
Weekly	10	11.9	30	9.4%	14	14	2	9.1	56	10.7	
Rarely	5	6	18	5.6	5	5	1	4.5	29	5.5	
Never	9	10.7	39	12.2	7	7	1	4.5	56	10.7	
4. Vegetable											
Daily	27	32.1	136	42.6	38	38	15	68.2	216	41.1	0.013^*^
2-3 times/week	31	36.9	76	23.8	32	32	5	22.7	144	27.4	
Weekly	11	13.1	61	19.1	14	14	1	4.5	87	16.6	
Rarely	9	10.7	12	3.8	8	8	0	0	29	5.5	
Never	6	7.1	34	10.7	8	8	1	4.5	49	9.3	
5. Fruits											
Daily	27	32.1	125	39.2	45	45	13	59.1	210	40	0.296
2-3 times/week	33	39.3	98	30.7	27	27	2	9.1	160	30.5	
Weekly	17	20.2	74	23.2	17	17	4	18.2	112	21.3	
Rarely	4	4.8	15	4.7	7	7	2	9.1	28	5.3	
Never	3	3.6	7	2.2	4	4	1	4.5	15	2.9	
6. Bread											
Daily	60	71.4	253	79.3	72	72	17	77.3	402	76.6	0.268
2-3 times/week	11	13.1	43	13.5	18	18	2	9.1	74	14.1	
Weekly	7	8.3	11	3.4	4	4	2	9.1	24	4.6	
Rarely	2	2.4	5	1.6	5	5	1	4.5	13	2.5	
Never	4	4.8	7	2.2	1	1	0	0	12	2.3	
7. Rice and other cereals											
Daily	62	73.8	232	72.7	71	71	19	86.4	384	73.1	0.983
2-3 times/week	16	19	60	18.8	19	19	2	9.1	97	18.5	
Weekly	4	4.8	19	6	8	8	1	4.5	32	6.1	
Rarely	1	1.2	2	0.6	1	1	0	0	4	0.8	
Never	1	1.2	6	1.9	1	1	0	0	8	1.5	
Total	84	16%	319	61%	100	19%	22	4%	525	100%	

The majority of female secondary students in all weight categories report eating snacks between meals (80.6%) and eating fast food (94%), with obese students eating fast food the highest percentage. The percentage of students who skipped meals was high (72%), particularly among those who were underweight. Daily consumption of hot drinks was 56.4%, while 65.5% never consumed energy drinks. All participants report a high daily consumption of sweets (52.8%), with the obese group showing the highest consumption. Most individuals across all weight categories report regular daily physical activity (56.2%), with obese individuals reporting slightly less. In terms of sleep hours, 18.9% of participants sleep less than six hours per day, and 32.8% sleep more than nine hours. Extensive use (≥5 hours) of electronic devices is common across all categories (57.5%), especially in overweight and obese individuals. Lastly, the findings of the chi-squared test conclude that eating fast food and consuming energy drinks show significant associations with weight status. Other habits, such as snacking, meal skipping, hot drink consumption, eating sweets, physical activity, sleep hours, and electronic device usage hours, do not have significant associations with weight status with a p-value less than 0.05 (Table 4).

**Table 4:** Association between participants' weight status and some eating patterns and lifestyles F: frequency; %: percentage; *: statistically significant

Variables	Underweight		Normal		Overweight		Obese		Total		P-value
	F	%	F	%	F	%	F	%	F	%	
1. Eating snacks between meals											
No	16	19	65	20.4	20	20	1	4.5	102	19.4	0.344
Yes	68	81	254	79.6	80	80	21	95.5	423	80.6	
2. Eating fast food											
No	9	10.7	16	5	3	3	0	0	28	5.3	0.045^*^
Yes	75	89.3	303	95	97	97	22	100	497	94	
3. Skipping meal											
No	21	25	93	29.2	30	30	3	13.6	147	28	0.386
Yes	63	75	226	70.8	70	70	19	86.4	378	72	
4. Drinking hot drinks											
Daily	46	54.8	182	57.1	54	54	14	63.6	296	56.4	0.446
2-3 times/week	15	17.9	68	21.3	27	27	1	4.5	111	21.1	
1 time/week	4	4.8	17	5.3	3	3	2	9.1	26	5	
Never	19	22.6	52	16.3	16	16	5	22.7	92	17.5	
5. Drinking energy drinks											
Daily	7	8.3	42	13.2	4	4	1	4.5	54	10.3	0.037^*^
2-3 times/week	12	14.3	31	9.7	7	7	1	4.5	51	9.7	
1 time/week	9	10.7	40	12.5	21	21	6	27.3	76	14.5	
Never	56	66.7	206	64.6	68	68	14	63.6	344	65.5	
6. Eating sweets											
Daily	48	57.1	158	49.5	55	55	16	72.7	277	52.8	0.379
2-3 times/week	25	29.8	105	32.9	35	35	4	18.2	169	32.2	
1 time/week	4	4.8	26	8.2	5	5	0	0	35	6.7	
Never	7	8.3	30	9.4	5	5	2	9.1	44	8.4	
6. Performing regular physical activity per week											
Daily	45	53.6	188	58.9	52	52	10	45.5	295	56.2	0.236
2-3 times/week	21	25	84	26.3	31	31	9	40.9	145	27.6	
1 time/week	7	8.3	30	9.4	10	10	3	13.6	50	9.5	
Never	11	13.1	17	5.3	7	7	0	0	35	6.7	
7. Number of sleep hours per day											
<6	19	22.6	57	17.9	18	18	5	22.7	99	18.9	0.596
7-8	38	45.2	151	47.3	56	56	9	40.9	254	48.4	
≥9	27	32.1	111	34.8	26	26	8	36.4	172	32.8	
8. Number of hours spent using mobile phones, tablets, or video games											
0	0	0	8	2.5	1	1	0	0	9	1.7	0.707
1-2	12	14.3	46	14.4	14	14	4	18.2	76	14.5	
3-4	22	26.2	90	28.2	22	22	4	18.2	138	26.3	
≥5	50	59.5	175	54.9	63	63	14	63.6	302	57.5	
Total	84	16%	319	61%	100	19%	22	4%	525	100%	

The ordinal logistic regression reveals that age and fast food consumption have significant effects on participants' weight status. Conversely, the variables of school grade, father's education, eating meat, and eating vegetables do not have significant effects on the participants' weight status, with a p-value of less than 0.05 (Table 5). 

**Table 5:** Predictors of weight status of the participants *: statistically significant

Variables	Odds ratio	Confidence interval (95%)		P-value
Age	1.318	0.078	0.474	0.006*
School grade	0.978	-0.445	0.401	0.917
Father's education	0.957	-0.224	0.136	0.630
Eating meat	1.026	-0.112	0.163	0.717
Eating vegetable	1.118	-0.025	0.247	0.109
Consuming fast food	2.233	0.033	1.573	0.041*

## Discussion

The study's findings indicate that two-fifths of female secondary school students show abnormalities in their weight status; 23% of them were overweight or obese, and 16% of them were underweight, which is considered a high rate. In comparison to previous studies, Alkoly et al. conducted a study among adolescents in Jeddah, Saudi Arabia, which concluded that the incidence of underweight is 43% and normal weight is 30% for female adolescents. Moreover, the combined prevalence of obesity and overweight was 27.1% [20]. The findings from additional research conducted in the United Arab Emirates to assess the nutritional health of female students indicate that 6.7% were classified as obese, 19.4% were categorized as overweight, 60.9% were considered to have a normal weight, and 13% were identified as underweight [21]. The prevalence of underweight, normal weight, overweight, and obesity among adolescent girls in Semnan, Iran, was 5.7%, 77.7%, 11.7%, and 4.7%, respectively [22]. These differences in our study's findings compared to other studies may be due to differences in culture, eating patterns, lifestyles, and economic status.

The chi-squared statistical test, with a p-value less than 0.05, revealed a statistical association between nutrition status and age group, grade, and father's education level. Those between the ages of 15 and 17 accounted for the majority of participants in the underweight (52.4%), normal weight (66.8%), overweight (69.1%), and obese (95.5%) categories, respectively. This may be due to the fact that most of the sample was from this age group. In India, a cross-sectional study conducted by Chandar et al. revealed a significant relationship between the nutritional status of adolescent girls and their age, with younger (10-14-year-old) adolescents exhibiting higher levels of malnutrition than their older counterparts [23]. A study carried out by Fatima et al. in the Kingdom of Saudi Arabia found that the highest rates of overweight and obesity were observed at the age of 17, while the highest rates of underweight were observed at 16 years old. Furthermore, there was no significant difference among adolescent girls who were obese across all age groups [24].

There is a statistical association between the consumption of meat and vegetables and weight status. The results showed that the participants who ate meat daily had few cases of weight gain and did not record any cases of obesity. Most people who have a normal weight do not eat meat or eat it rarely. As for vegetables, two-fifths of the participants ate it daily, and this was considered the largest percentage. A study conducted in a rural region of West Bengal discovered that approximately 50% of the participants periodically consumed fruits, vegetables, eggs, and meat [25]. Another study conducted in Adama City, Ethiopia, found that school adolescent females who had a higher diet diversity score (DDS) were more likely to have normal BMI-for-age Z scores, in contrast to those with lower diet diversity scores [26]. Thus, diet diversity affects weight; including a wide variety of foods in one's diet typically promotes improved nutritional well-being and can aid in keeping a healthy body weight.

Several unhealthy eating behaviors and lifestyles have emerged in Iraq and other countries in recent decades, especially among adolescents. In this study, we focused on the most common ones. Our study findings revealed that the majority of female secondary students, irrespective of their weight categories, reported snacking in between meals. Additionally, obese students consume the highest proportion of fast food, and there is a significant association between fast food consumption and participants' weight status. In a study of Saudi Arabian adolescents, 90% of the females consumed fast food [20]. Another study on eating habits conducted among Nigerian urban female adolescents concluded that over three-quarters (76%) of them typically consumed fast food and soft drinks [12]. About three-quarters of the female adolescents skipped meals during the day. Studies from various other developing countries have documented similar findings, so this is not surprising [27,28].

An ordinal logistic regression was conducted for the variables that show a significant association in the chi-squared test. The results of the logistic regression indicate that the odds ratio significantly influences the participants' weight status, with age 1.318 (CI 95%: 0.078-0.474) and fast food consumption 2.233 (CI 95%: 0.033-1.573).

We must consider a few limitations of our study. Firstly, our study solely focused on adolescent girls, thereby limiting the generalizability of the results to both genders. Secondly, the sampling location limits the findings, as it only included urban female secondary schools and could not be generalized to rural female secondary schools.

## Conclusions

The study concludes that approximately a quarter of the female secondary school students were overweight or obese and about one-sixth of them were underweight. One-third of them never ate meat, and the same rate ate it rarely. Most of the participants had snacks between meals and frequently ate fast food. On the other hand, the highest percentage of them were skipping meals, particularly among those who were underweight. A school-based nutrition program should be applied in Iraqi schools in order to improve the nutritional status and eating behaviors of the students.
